# Health inequality: a longitudinal study on geographic variations in lung cancer incidence and mortality in Taiwan

**DOI:** 10.1186/s12889-020-09044-2

**Published:** 2020-06-17

**Authors:** Jason C. Hsu, Yu-Chi Tseng, Sheng-Mao Chang, Yang-Cheng Lee, Peng-Chan Lin, Hone-Jay Chu

**Affiliations:** 1 International Ph.D. Program in Biotech and Healthcare Management, College of Management, Taipei Medical University, Taipei, Taiwan; 2 Institute of Clinical Pharmacy and Pharmaceutical Sciences, College of Medicine, National Cheng Kung University, Tainan, Taiwan; 3 Department of Statistics, College of Management, National Cheng Kung University, Tainan, Taiwan; 4 Department of Internal Medicine, Tainan Municipal Hospital, Tainan, Taiwan; 5 Department of Internal Medicine, National Cheng Kung University Hospital, College of Medicine, National Cheng Kung University, Tainan, Taiwan; 6 Department of Geomatics, National Cheng Kung University, Tainan, Taiwan

**Keywords:** Geographical variation, Lung cancer, Incidence, Mortality

## Abstract

**Background:**

This study is aimed toward an analysis of the variations in lung cancer incidence and mortality, adjusted by population factors (age, gender, and year), between administrative areas.

**Methods:**

This is a retrospective study, using 2005–2014 data in each administrative area from the Taiwan Cancer Registry database organized by the Health Promotion Administration. The yearly age-standardized (overall) and crude (stratified by gender and age) incidence/mortality (and their growth rates) for each administrative area were collected and calculated. We used a mixed model to analyze the repeated measurements of yearly incidence and mortality rates and used general linear regression to analyze their growth rates.

**Results:**

It was found that male and elderly populations had significantly higher lung cancer incidence and mortality in Taiwan. After adjusting for gender, age, and calendar year, there were no significant variations in incidence among the administrative areas, while the mortality in Yilan County was significantly higher than that in Taipei City (the capital city of Taiwan). On the other hand, the incidence in the female and younger population and mortality growth rates were higher. The incidence growth rate in Keelung City was significantly lower than that in Taipei City, while there were no significant variations in mortality growth rate among administrative areas.

**Conclusions:**

This study found an inequality in the lung cancer burden among cities in Taiwan, which can serve as the basis for future resource allocations for lung cancer prevention and treatment in Taiwan.

## Background

According to the results of the GLOBOCAN 2018 project conducted by the World Health Organization, lung cancer has become one of the leading causes of mortality worldwide. The age-standardized incidence of lung cancer ranks number one among all cancers for the global population (22.5 per 100,000) for both sexes combined, and it ranked number one for men (31.5 per 100,000) and number two for women (14.6 per 100,000) in 2018. The age-standardized mortality of lung cancer is number one among all cancers for the global population (18.6per 100,000) for both sexes combined, and it ranks number one for men (27.1 per 100,000) and number two for women (11.2 per 100,000) in 2018. The ranking for lung cancer incidence and mortality is similar in Taiwan [1].

In the last few years, several articles have been devoted to the study of geographic variations in the incidence and mortality of lung cancer between and within various countries or regions. Some articles were aimed toward identifying the areas in countries/states that had a need for more attention due to the higher incidence [2–7] or mortality [7–11] of lung cancer. These studies found that the incidence of cancer is higher in the eastern and southern states of the US [5], and that it is the highest in the southeastern region of Canada [4]. In terms of mortality, compared to the northwest, mortality is higher in the north, east, central, and northeast [risk ratio (RR)] = 2.44 in China [10]. In addition, several studies have focused on tobacco-related countries [12] or areas [13, 14], for which the results show that the incidence and mortality of lung cancer are highest in countries where smoking uptake began the earliest, such as those in North America and Europe, even though the rates are now decreasing in most of these countries [12]. Furthermore, some articles have explored the association between industrial cities [15] or cities with coal mine exposure [16] and the incidence and mortality of lung cancer, and significant relationships have been observed.

Previous studies have shown geographic variations in lung cancer incidence and mortality stratified by patient age, gender, and year. However, previous literature on this topic has mostly focused on static levels of disease burden, thus lacking analyses of dynamic growth rates among administrative areas. In addition, only geographic variations without adjustment for population factors have been reported. This study is aimed toward addressing these gaps by examining the dynamic growth of both lung cancer incidence and mortality in various administrative areas in Taiwan in order to determine which areas will need more attention in the future. Furthermore, in order to obtain more precise conclusions, adjustments for age, gender, and calendar year are considered to analyze the geographic variations among administrative areas in Taiwan.

## Methods

### Data source

A retrospective design was adopted in this study, and yearly data from the Taiwan Cancer Registry database through the online interactive inquiry system developed by the Health Promotion Administration [17], the Ministry of Health and Welfare [18] were used to collect lung cancer data in the various administrative areas from the period 2005 to 2014 (10 years). The database includes the crude and age-standardized incidence and mortality of cancer stratified according to gender, age, and calendar year, and other relevant information. The data for people ≥20 years old were observed.

### Measurements

To identify the area with both the highest incidence and mortality of lung cancer in Taiwan, considering both gender and all age, we collected and calculated yearly age-standardized incidence and mortality of lung cancer for 19 administrative areas (excluding the outer islands) from 2005 to 2014 using the world standard population in 2000 and the following formulas:

*Age-standardized incidence = Σ (Incidence by age × Standard population for the age group) ÷ Σ (Standard population for a specific age group) × 100,000;*

*Age-standardized mortality = Σ (Mortality by age × Standard population for the age group) ÷ Σ (Standard population for a specific age group) × 100,000.*

In addition, we also calculated the age-standardized incidence and mortality growth rates (2005–2014) of lung cancer in each administrative area based on their regression equations.

Furthermore, to analyze the geographic variations among the administrative areas in Taiwan taking adjustments for age, gender, and calendar year into consideration, the crude incidence and mortality, stratified by administrative area, age, gender, and calendar year were also collected for the multivariate analysis. We investigated the main effects of administrative area on incidence and mortality, adjusted by gender, age, and calendar year. The annual growth rates (2005–2014) of incidence and mortality were calculated by using the slope of the regression line for each group with the same administrative area, age, and gender.

In 2010, many administrative regions were merged together in Taiwan. The data for each administrative region in this study from 2005 to 2014 was based on the combined administrative regions. Basic information for all administrative areas in Taiwan is shown in the Additional file 1 [19, 20].

### Statistical approach

A geographic map and four-quadrant scatter plot were used to visualize the geographic variations in the lung cancer burden among the various administrative areas. We first showed the age-standardized incidence and mortality of lung cancer in each area in 2014 and their growth rates (2005–2014) using geographic maps. Then, to identify the administrative areas needing more attention, we applied the four-quadrant scatter plot method and put the age-standardized incidence and mortality for each area together in the same plane. The means of the incidence/mortality (and their growth rates) for all administrative areas were set to the cut-off point for separating high/low incidence (mortality and their growth rate).

For the analysis of the association between administrative area and incidence/mortality of lung cancer, since the data used in this study was the data for each administrative area from 2005 to 2014 (10 years), the annual repeated measurement data for the same area was independent. Therefore, a regular linear regression analysis was no longer valid. Instead, the mixed model approach, a statistical model containing both fixed effects and random effects, properly takes this independency into account. It has been applied in many disciplines where multiple correlated measurements are made on each unit of interest. In the data analysis, we posited the compound symmetry correlation structure to model the annual incidence rates and the annual mortality rates. As for the association between administrative area and incidence/mortality growth rates, a general linear regression was applied. The IBM SPSS Statistics 25 package software was used for all data processing and statistical analyses.

## Results

**Fig. 1:**
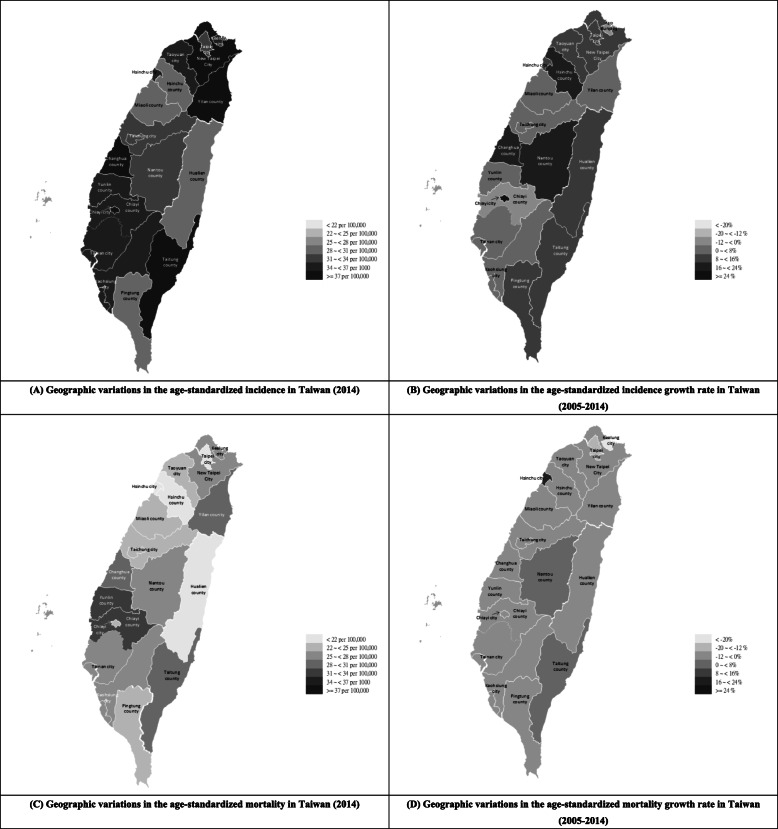
Geographic variations in age-standardized incidence, mortality, and their growth rates in Taiwan. **a** Geographic variations in the age-standardized incidence in Taiwan (2014); **b** Geographic variations in the age-standardized incidence growth rate in Taiwan (2005–2014); **c** Geographic variations in the age-standardized mortality in Taiwan (2014); **d** Geographic variations in the age-standardized mortality growth rate in Taiwan (2005–2014)

The yearly age-standardized incidence, mortality (2014), and growth rates (2005–2014) of lung cancer in each administrative area in Taiwan were collected and calculated (Table 1), and the previous data was visualized with the geographic maps shown in Fig. 1 (made by using Microsoft office excel 2003 software). Changhua County had the highest age-standardized incidence (40.28 per 100,000) in 2014, followed by Yilan County (39.63 per 100,000) and New Taipei City (39.29 per 100,000). Chiayi City had the highest incidence growth rate (25.81%) from 2005 to 2014, followed by Hsinchu County (23.13%) and Nantou County (22.83%). Yunlin County had the highest age-standardized mortality (32.4 per 100,000) in 2014, followed by Chiayi County (32.1 per 100,000) and Yilan County (30.2 per 100,000). Hsinchu City had the highest mortality growth rate (17.38%) from 2005 to 2014, followed by Nantou County (3.73%) and Taitung County (0.67%).

**Table 1:** Yearly age-standardized incidence, mortality (2014), and their growth rates (2005–2014) for lung cancer in each administrative area in Taiwan

Administrative Areas	Age-standardized Incidence (2014) (per 100,000)	Age-standardized Incidence growth rate (2005–2014) (%)	Age-standardized Mortality (2014) (per 100,000)	Age-standardized Mortality growth rate (2005–2014) (%)
Taipei City	33.36	10.83	20.3	−14.31
Keelung City	35.33	−11.08	27.6	−25.63
New Taipei City	39.29	12.68	25.2	−9.60
Yilan County	39.63	2.60	30.2	−9.16
Taoyuan City	34.83	12.22	24.8	−10.21
Hsinchu City	34.64	11.21	21	17.38
Hsinchu County	30.6	23.13	20.7	−2.94
Miaoli County	28.74	5.62	23	−11.93
Taichung City	33.69	6.03	24.6	−7.26
Changhua County	40.28	17.24	29.9	−5.53
Nantou County	31.43	22.83	25.4	3.73
Yunlin County	35.66	3.00	32.4	−1.04
Chiayi City	38.47	25.81	24.5	−6.45
Chiayi County	35.04	−0.96	32.1	−6.36
Tainan City	34.76	2.30	26	−3.64
Kaohsiung City	36.67	7.21	26.7	−6.66
Pingtung County	30.62	8.44	24.9	−1.96
Hualien County	28.73	8.22	21.7	−9.92
Taitung County	37.87	15.75	28.4	0.67

The age-standardized incidence and mortality growth rates (2005–2014) of lung cancer in each administrative area were calculated based on their regression equations instead of their absolute rates

Incidence growth rate (2005–2014) = [(incidence in 2014)–(incidence in 2005)]÷(incidence in 2005)*100%;

Mortality growth rate (2005–2014) = [(mortality in 2014)–(mortality in 2005)]÷(mortality in 2005)*100%

**Fig. 2:**
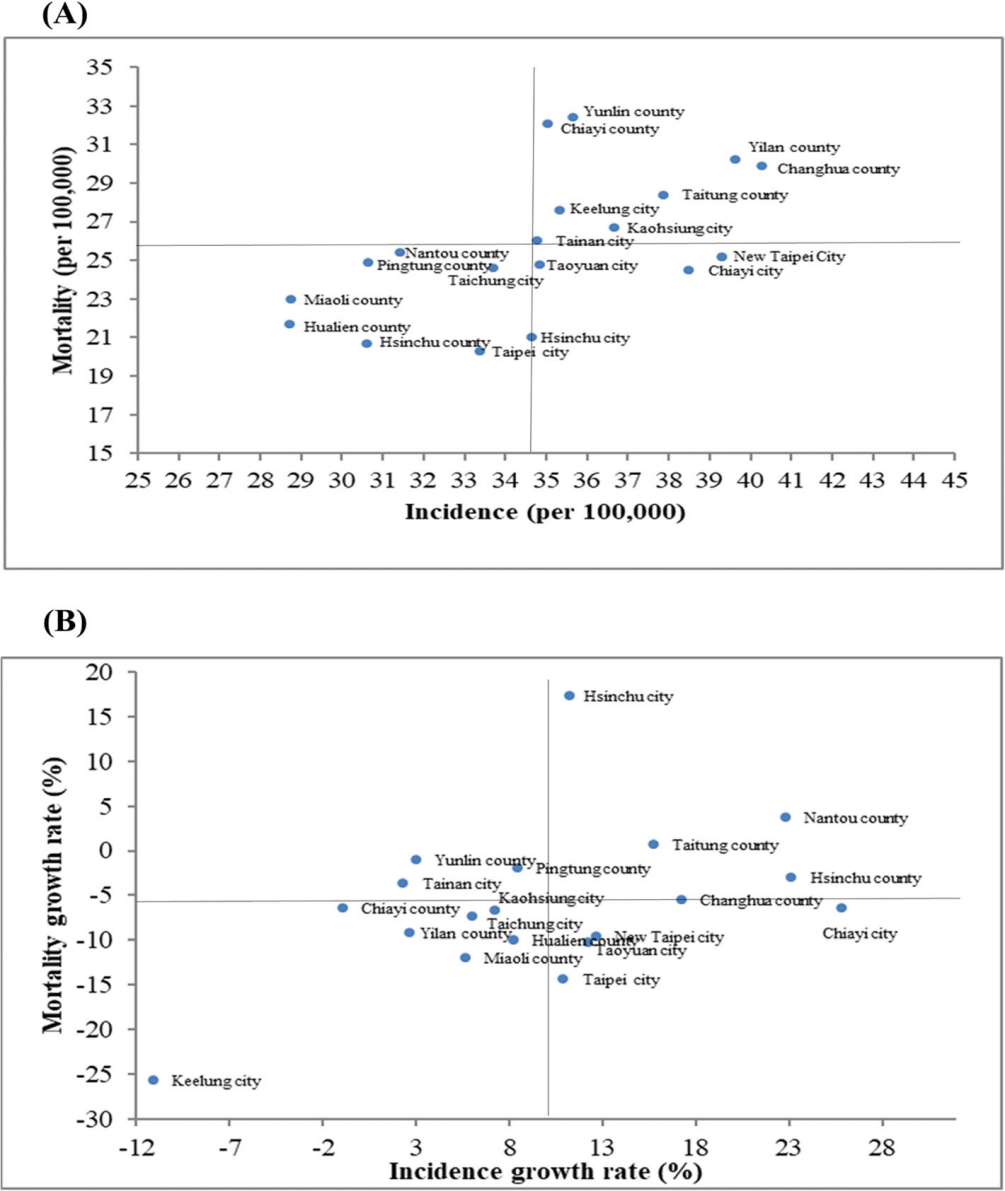
Four-quadrant scatter plot. **a** age-standardized incidence and mortality (2014); **b** age-standardized incidence and mortality growth rates (2005–2014)

A four-quadrant scatter plot based on both age-standardized incidence and mortality is provided in Fig. 2a. We calculated the distance from the incidence-mortality (growth rate) point of each administrative area to the origin on the four-quadrant scatter plots (Table 2). All administrative areas were divided into 3 categories: (1) high incidence and high mortality: Changhua County (distance to the origin = 6.93), Yunlin County (6.71), Yilan County (6.62), Chiayi County (6.35), Taitung County (4.11), Kaohsiung City (2.17), Keelung City (1.94), and Tainan City (0.25); (2) high incidence but low mortality: New Taipei City (4.61), Chiayi City (3.96) and Taoyuan City (0.96); (3) low incidence and low mortality: Hualien County (7.23), Miaoli County (6.58), Hsinchu County (6.52), Taipei City (5.62), Hsinchu City (4.76), Pingtung County (4.19), Nantou County (3.31), and Taichung City (1.55).

**Table 2:** The distance from the incidence/mortality (growth rate) point to the origin on the four-quadrant scatter plots

Administrative Areas	The distance from incidence/mortality point to the means of incidence and mortality	The distance from incidence/mortality growth rate to the means of incidence growth rates and mortality growth rates
Taipei City	5.62	8.56
Keelung City	1.94	28.65
New Taipei City	4.61	4.84
Yilan County	6.62	7.78
Taoyuan City	0.96	5.08
Hsinchu City	4.76	23.27
Hsinchu County	6.52	13.80
Miaoli County	6.58	7.30
Taichung City	1.55	3.88
Changhua County	6.93	7.61
Nantou County	3.31	16.30
Yunlin County	6.71	8.19
Chiayi City	3.96	16.19
Chiayi County	6.35	10.61
Tainan City	0.25	7.66
Kaohsiung City	2.17	2.56
Pingtung County	4.19	4.05
Hualien County	7.23	4.33
Taitung County	4.11	8.93

The means of the incidence / mortality (and their growth rates) for all administrative areas were set to the cut-off point separating high/low incidence (mortality and their growth rate)

The distance from the incidence / mortality (growth rate) point for each administrative area to the origin (mean) on the four-quadrant scatter plots was calculated

Fig. 2b shows the four-quadrant scatter plot based on both age-standardized incidence growth rates and mortality growth rates. All administrative areas were divided into 4 categories: (1) high incidence growth rate and high mortality growth rate: Hsinchu City (distance to the origin = 23.27), Nantou County (16.3), Hsinchu County (13.8), Taitung County (8.93), and Changhua County (7.61); (2) high incidence growth rate but low mortality growth rate: Chiayi City (16.19), Taipei City (8.56) and Taoyuan City (5.08), and New Taipei City (4.84); (3) low incidence growth rate but high mortality growth rate: Yunlin County (8.19), Tainan City (7.66), and Pingtung County (4.05); (4) low incidence growth rate and low mortality growth rate: Keelung City (28.65), Hsinchu City (23.27), Chiayi County (10.61), Yilan County (7.78) Miaoli County (7.3), Hualien County (4.33), Taichung City (3.88), and Kaohsiung City (2.56)

Table 3 shows the statistical results for the association between administrative area and incidence/mortality of lung cancer, adjusted by age, gender, and calendar year. The male and elderly populations had a significantly higher incidence and mortality of lung cancer in Taiwan. After adjusting for gender, age, and calendar year, there were no significant variations in incidence among the administrative areas, while the mortality in Yilan County was significantly higher than that in Taipei City (the capital city of Taiwan). On the other hand, female and younger population’s incidence and mortality growth rates were higher. The incidence growth rate in Keelung City was significantly lower than that in Taipei City, while there were no significant variations of mortality growth rate between administrative areas.

**Table 3:** Statistical results of the association between administrative area and incidence/mortality of lung cancer

Factors	Incidence				Mortality			
	Estimate	Sig.	95% Confidence Interval		Estimate	Sig.	95% Confidence Interval	
			Lower Bound	Upper Bound			Lower Bound	Upper Bound
**Intercept**	**34.05**	**0.03**	**2.67**	**65.44**	**34.70**	**0.04**	**2.09**	**67.31**
**Administrative Areas**								
Taipei City	0.00				0.00			
Keelung City	22.02	0.20	−11.40	55.45	24.63	0.17	−10.21	59.46
New Taipei City	27.50	0.11	−5.93	60.92	13.37	0.45	−21.47	48.20
Yilan County	28.94	0.09	−4.49	62.36	**35.79**	**0.04**	0.95	70.62
Taoyuan City	10.41	0.54	−23.01	43.83	9.01	0.61	−25.83	43.84
Hsinchu City	−6.61	0.70	−40.03	26.81	−7.57	0.67	−42.40	27.27
Hsinchu County	−18.85	0.27	−52.27	14.57	−3.98	0.82	−38.82	30.85
Miaoli County	−20.17	0.24	−53.60	13.25	−6.26	0.72	−41.10	28.57
Taichung City	6.17	0.72	−27.25	39.60	10.38	0.56	−24.45	45.22
Changhua County	15.48	0.36	−17.94	48.90	26.18	0.14	−8.66	61.01
Nantou County	−17.84	0.30	−51.27	15.58	2.24	0.90	−32.59	37.08
Yunlin County	7.14	0.68	−26.28	40.56	28.82	0.11	−6.01	63.66
Chiayi City	6.08	0.72	−27.34	39.50	2.42	0.89	−32.41	37.26
Chiayi County	14.65	0.39	−18.77	48.08	32.60	0.07	−2.24	67.43
Tainan City	8.91	0.60	−24.51	42.34	14.01	0.43	−20.83	48.84
Kaohsiung City	3.45	0.84	−29.97	36.87	8.55	0.63	−26.29	43.38
Pingtung County	−13.56	0.43	−46.98	19.87	2.54	0.89	−32.29	37.38
Hualien County	−8.80	0.61	−42.22	24.63	2.41	0.89	−32.42	37.25
Taitung County	6.17	0.72	−27.25	39.60	27.72	0.12	− 7.12	62.55
**Gender**								
Male	0.00				0.00			
Female	**− 86.37**	**0.00**	−97.22	−75.53	**− 88.74**	**0.00**	−100.04	−77.44
**Age**								
20–24	0.00				0.00			
25–29	0.46	0.98	−28.23	29.15	0.18	0.99	−29.72	30.08
30–34	2.34	0.87	−26.35	31.03	1.47	0.92	−28.44	31.37
35–39	5.87	0.69	−22.82	34.56	3.50	0.82	−26.40	33.40
40–44	14.44	0.32	−14.25	43.13	8.12	0.59	−21.78	38.02
45–49	**25.74**	**0.08**	−2.95	54.43	15.30	0.32	−14.60	45.21
50–54	**43.42**	**0.00**	14.73	72.11	27.20	0.08	−2.70	57.10
55–59	**71.79**	**0.00**	43.10	100.48	**46.92**	**0.00**	17.01	76.82
60–64	**109.01**	**0.00**	80.32	137.70	**76.48**	**0.00**	46.58	106.38
65–69	**171.19**	**0.00**	142.50	199.88	**125.13**	**0.00**	95.23	155.03
70–74	**250.64**	**0.00**	221.95	279.33	**202.97**	**0.00**	173.07	232.87
74–49	**328.43**	**0.00**	299.75	357.12	**294.07**	**0.00**	264.16	323.97
80–84	**369.81**	**0.00**	341.12	398.50	**373.02**	**0.00**	343.12	402.92
> 85	**351.26**	**0.00**	322.57	379.95	**416.23**	**0.00**	386.33	446.13
**Year**								
2005	0.00				0.00			
2006	2.44	0.41	−3.33	8.22	−0.04	0.99	−5.84	5.75
2007	2.22	0.45	−3.55	8.00	4.91	0.10	−0.89	10.70
2008	0.94	0.75	−4.84	6.71	−4.10	0.17	−9.90	1.69
2009	**11.80**	**0.00**	6.03	17.58	−3.97	0.18	−9.76	1.82
2010	**10.89**	**0.00**	5.11	16.66	−1.20	0.68	−7.00	4.59
2011	**11.06**	**0.00**	5.28	16.84	1.27	0.67	−4.52	7.06
2012	**5.84**	**0.05**	0.06	11.62	−4.64	0.12	−10.43	1.15
2013	5.49	0.06	−0.28	11.27	**− 7.09**	**0.02**	−12.88	−1.30
2014	4.37	0.14	−1.41	10.14	−4.81	0.10	−10.60	0.98
−2 Restricted Log Likelihood	57,527.295				57,620.812			
Akaike’s Information Criterion (AIC)	57,541.295				57,634.812			
Schwarz’s Bayesian Criterion (BIC)	57,587.294				57,680.811			

Model: Mixed model

Repeated measurement: compound symmetry

Distribution: Gamma regression

Gender and age were adjusted

Table 4 shows that the incidence and mortality growth rates in the female and younger populations were higher. Using Taipei City as the reference, the incidence growth rate of lung cancer was significantly lower in Keelung City after adjusting age and gender. In terms of the mortality growth rate of lung cancer, there were no significant variations found between administrative areas.

**Table 4:** Statistical results for the association between administrative area and incidence/mortality growth rate of lung cancer (general linear regression, adjusted by gender and age)

	Incidence Growth Rate					Mortality Growth Rate				
	SS	DF	MS	F	Sig.	SS	DF	MS	F	Sig.
Intercept	2097.424	1	2097.424	9.04	0.003	2641.545	1	2641.545	9.873	0.002
city	7024.615	18	390.256	1.682	**0.039**	3646.008	18	202.556	0.757	0.751
gender	3083.842	1	3083.842	13.292	**0.000**	2178.501	1	2178.501	8.143	**0.005**
age	7708.991	13	592.999	2.556	**0.002**	44,702.152	13	3438.627	12.853	**0.000**
**Factors**	**Incidence growth rate**					**Mortality growth rate**				
	**Estimate**	**Sig.**		**95% Confidence Interval**		**Estimate**	**Sig.**	**95% Confidence Interval**		
				**Lower Bound**	**Upper Bound**			**Lower Bound**	**Upper Bound**	
**Administrative Areas**										
Taipei City	0.00					0.00				
Keelung City	**−8.71**	**0.03**		−16.71	−0.72	−5.16	0.24	−13.74	3.43	
New Taipei City	4.44	0.28		−3.56	12.44	2.45	0.58	−6.14	11.04	
Yilan County	−1.87	0.65		−9.87	6.13	−1.60	0.71	−10.19	6.99	
Taoyuan City	5.43	0.18		−2.57	13.43	3.31	0.45	−5.28	11.90	
Hsinchu City	3.15	0.44		−4.85	11.15	1.43	0.74	−7.16	10.02	
Hsinchu County	0.95	0.82		−7.05	8.94	4.45	0.31	−4.14	13.04	
Miaoli County	−0.41	0.92		−8.41	7.58	1.97	0.65	−6.62	10.56	
Taichung City	−1.29	0.75		−9.29	6.71	1.49	0.73	−7.10	10.08	
Changhua County	1.06	0.80		−6.94	9.06	1.24	0.78	−7.35	9.82	
Nantou County	5.73	0.16		−2.26	13.73	4.57	0.30	−4.02	13.16	
Yunlin County	−2.99	0.46		−10.99	5.01	3.03	0.49	−5.55	11.62	
Chiayi City	3.79	0.35		−4.21	11.79	−3.44	0.43	−12.03	5.15	
Chiayi County	−2.09	0.61		−10.09	5.91	5.23	0.23	−3.36	13.82	
Tainan City	−2.19	0.59		−10.19	5.81	2.51	0.57	−6.08	11.10	
Kaohsiung City	−0.72	0.86		−8.72	7.27	−0.05	0.99	−8.64	8.54	
Pingtung County	−0.19	0.96		−8.19	7.81	2.26	0.61	−6.33	10.85	
Hualien County	1.77	0.67		−6.23	9.76	4.10	0.35	−4.49	12.69	
Taitung County	6.93	0.09		−1.07	14.93	1.41	0.75	−7.18	10.00	
**Gender**										
Male	0.00					0.00				
Female	**4.82**	**0.00**		2.22	7.41	**4.05**	**0.01**	1.26	6.83	
**Age**										
20–24	0.00					0.00				
25–29	0.00	1.00		−6.87	6.86	0.05	0.99	−7.32	7.43	
30–34	−0.10	0.98		−6.97	6.77	−0.14	0.97	−7.51	7.24	
35–39	0.09	0.98		−6.78	6.95	−0.13	0.97	−7.50	7.24	
40–44	1.40	0.69		−5.46	8.27	0.22	0.95	−7.16	7.59	
45–49	4.01	0.25		−2.86	10.87	0.81	0.83	−6.57	8.18	
50–54	4.17	0.23		−2.70	11.03	1.15	0.76	−6.23	8.52	
55–59	6.72	0.06		−0.14	13.59	−1.29	0.73	−8.67	6.08	
60–64	1.58	0.65		−5.28	8.45	−0.41	0.91	−7.78	6.96	
65–69	5.61	0.11		−1.26	12.47	−4.49	0.23	−11.86	2.89	
70–74	−1.76	0.62		−8.63	5.11	**−11.14**	**0.00**	−18.51	−3.77	
74–49	**−7.63**	**0.03**		−14.49	−0.76	**−21.42**	**0.00**	−28.80	−14.05	
80–84	5.01	0.15		−1.85	11.88	**−15.20**	**0.00**	−22.57	−7.83	
> 85	**7.16**	**0.04**		0.29	14.02	**20.26**	**0.00**	12.88	27.63	

*SS* sum of square; *DF* degree of freedom; *MS* Mean square; *Sig.* significant

*Sig.* significant

## Discussion

The incidence and mortality of lung cancer appear to have been geographically different in Taiwan in 2014 (Fig. 1). Incidence and mortality represent the severity of the disease burden, and their growth rate represents the rate of deterioration of the disease burden, both of which are important. However, previous research has generally focused on a comparison of the static level of the disease burden, but the dynamic speed (growth rate) of the disease burden is lacking in these studies. This study is an attempt to determine the differences between potential hazardous areas by comparing the static and dynamic features of geographic locations and by further identifying the administrative areas that are of high concern. Furthermore, the previous literature on the geographical differences in the incidence and mortality of lung cancer in Taiwan has statistically fewer corrections for other populations and other factors, which may have led to less objective results. Thus, this study included gender, age, and calendar year to improve the accuracy of the data analysis and to increase the applicability of the research results in future lung cancer policies.

This study first identified the administrative areas needing more attention by analyzing the age-standardized incidence, mortality of lung cancer, and their growth rates across all administrative areas of Taiwan in 2014. As far as the age-standardized incidence is concerned, it is assumed that there are not many differences in the medical diagnostic technologies and diagnostic penetration rates in the administrative areas of Taiwan. People living in administrative areas with high incidence may have higher lung cancer-related risk factors, for example, biological inheritance [21], air pollution (PM2.5) [22], asbestos [23, 24], or tobacco use [25]. Recent studies have indicated that new cancer cases can be avoided by reducing exposure to known environmental and lifestyle risk factors [26, 27]. We found that the highest age-standardized incidence occurred in Changhua County (Fig. 1), and the highest age-standardized incidence growth rates occurred in Chiayi City. Therefore, these areas need special attention to determine possible risk factors. On the other hand, age-standardized mortality represents the level of disease burden and medical capacity in a given area. We found that the highest age-standardized mortality of lung cancer was in Yunlin County and Chiayi County, and the highest age-standardized mortality growth rate of lung cancer was in Hsinchu City. In general, since all of the above areas are located in the central region of Taiwan, the central region of Taiwan is of high concern. It is thus necessary to further develop strategies for the prevention and treatment of lung cancer in the central region of Taiwan.

In addition, when we looked at the age-standardized incidence and mortality together (Fig. 2a), we generally found that the higher the age-standardized incidence, the higher the age-standardized mortality. In the four-quadrant scatter plot, Changhua County, which is located in the high incidence and high mortality quadrants, is the furthest from the origin, thus requiring special attention. In contrast, Hualien County, which is located in the low incidence and low mortality quadrants, is the furthest from the origin, which might be the safest place. In the future, it would be worthwhile to explore whether these low-incidence administrative areas are due to the presence of fewer lung cancer risk factors, such as low air pollution and low smoking rates. The results of this study can serve as a reference for future national cancer control policies.

Similarly, when we look at the age-standardized incidence and mortality growth rate together (Fig. 2b), in the four-quadrant scatter plot, Hsinchu City, which is in the high incidence growth rate and high mortality growth rate quadrants, is the furthest from the origin, thus requiring more attention. Conversely, Keelung City, which is located in the low incidence and low mortality growth rate quadrants, is the furthest from the origin, and it is the safest and does not require serious attention.

Based on the above results, we found that the highest age-standardized incidence of lung cancer in Taiwan in 2014 was in Changhua County. However, after adjusting for gender, age, and calendar year, we did not find any significant variations among the administrative areas, including Changhua County (Table 3). Similarly, the highest age-standardized incidence growth rate of lung cancer in 2014 was in Chiayi City; however, considering the adjustments, Chiayi City’s incidence growth rate was not significantly higher than that of Taipei, but Keelung City’s incidence growth rate was significantly lower than that of Taipei.

On the other hand, the highest lung cancer age-standardized mortality of in Taiwan in 2014 occurred in Yunlin County. However, after adjusting for gender, age and calendar year, only Yilan County had a significantly higher incidence growth rate as compared to that in Taipei City. As for mortality growth rate, even though the age-standardized mortality growth rate in Hsinchu City was the highest, after adjustment by gender and age, no significant variations were found among the administrative areas.

The above findings can serve as a reference for resource allocation for the government’s future lung cancer prevention and control policies, especially the results obtained using the adjusted data. The results of this study show the incidence rates of lung cancer to be noteworthy in Yilan County and New Taipei City. It is recommended that the government’s future environmental protection policies and national smoke prevention and control policies receive higher attention in these areas. Our results show that Keelung City had a high incidence, but its incidence growth rate was significantly lower than that for Taipei, which means that the incidence of lung cancer had improved in Keelung City.

On the other hand, the possible causes of high lung cancer mortality and its associated growth rate may be related to the local medical service resources in addition to the local disease burden (incidence) [28]. It was found here that only Yilan County had a significantly higher mortality than Taipei City. Therefore, it is suggested that the government should first strengthen its lung cancer medical resources in the future, including the availability, accessibility, and affordability of medical treatment for the disease.

There are some limitations to this study. First, this study was an effort to examine the geographic variations in lung cancer incidence and mortality in Taiwan. We obtained the lung cancer incidence and mortality data from the Taiwan Cancer Registration Database compiled by the Health Promotion Administration. The database has data regarding overall lung cancer, but it has no information about subtype and staging of lung cancer. It is recommended that follow-up researchers collect staging data to obtain more specific and thus more rigorous results. Second, there are urban-rural differences associated with medical resource distribution. Urban-rural-related data, such as urban hierarchy, population density, income level, industrial structure, and other indicators, might explain the variations in medical resources across administrative areas, which is considered relevant to mortality. Urban-rural differences could be used as another factor for adjustment for future studies. Third, many of the areas with low growth rates are considered to have high rates, and vice versa. When rates are low, small changes can lead to large percentage increases (growth). Furthermore, this study did not explore other factors that affect the lung cancer burden, including smoking and air pollution. It is suggested that other data contributing to lung cancer occurrence could be collected and analyzed in the future.

## Conclusions

This study provided an analysis of geographic variations in the severity of the disease burden and the rate of deterioration based on geographic differences in lung cancer incidence, mortality, and their respective growth rates. Overall, our research findings suggest that the government should strengthen the prevention and treatment policies and resources in Yilan County because its incidence (non-significant) and mortality (significant) were higher than those in Taipei City. Specifically, Keelung City had the lowest growth rates of incidence (significant) and mortality (non-significant), which can be the benchmark. The results of this study help lead to an understanding of health inequalities, help identify the focus for geographic areas (areas needing more attention) in which lung cancer is prevalent, and serve as the basis for the allocation of resources for the future prevention and treatment of lung cancer in Taiwan.

## Supplementary information

**Additional file 1.**

## Data Availability

The authors obtained nationwide, yearly data for lung cancer in the various administrative areas, from 2005 to 2014, from the Taiwan Cancer Registry database through the online interactive inquiry system (website: https://cris.hpa.gov.tw/) made by Health Promotion Administration in Taiwan. The data was publicly available.

## Abbreviations

RR: Risk ratio
PM2.5: Particulate matter 2.5
SS: Sum of square
DF: Degree of freedom
MS: Mean square
Sig: significant
