# Supplementary material for: Health inequality: a longitudinal study on geographic variations in lung cancer incidence and mortality in Taiwan
Source: BMC Public Health. 2020 Jun 17;20:951. doi: 10.1186/s12889-020-09044-2 (PMC7301436; doi:10.1186/s12889-020-09044-2)
Supplement: Supplementary file 1 — Additional file 1. [file 12889_2020_9044_MOESM1_ESM.doc]

**Appendix. Basic information for all administrative areas in Taiwan**

| **Administrative Areas** | **Population (December 2014)** | **Land area size (Square kilometer)** |
| --- | --- | --- |
| Taipei City | 2,702,315 | 272 |
| Keelung City | 373,077 | 133 |
| New Taipei City | 3,966,818 | 2,053 |
| Yilan County | 458,777 | 2,144 |
| Taoyuan City | 2,058,328 | 1,221 |
| Hsinchu City | 431,988 | 104 |
| Hsinchu County | 537,630 | 1,428 |
| Miaoli County | 567,132 | 1,820 |
| Taichung City | 2,719,835 | 2,215 |
| Changhua County | 567,132 | 1,074 |
| Nantou County | 567,132 | 4,106 |
| Yunlin County | 705,356 | 1,291 |
| Chiayi City | 270,883 | 60 |
| Chiayi County | 524,783 | 1,904 |
| Tainan City | 1,884,284 | 2,192 |
| Kaohsiung City | 2,778,992 | 2,948 |
| Pingtung County | 847,917 | 2,776 |
| Hualien County | 333,392 | 4,629 |
| Taitung County | 224,470 | 3,515 |
